# Dental Erosion Management: From Remineralization to Emerging Regenerative Approaches—A Narrative Review

**DOI:** 10.3390/biomimetics11020107

**Published:** 2026-02-03

**Authors:** Ruvienath Daham Weerasinghe Rajapaksa, Yu-Ching Wang, Yong Chen Chin, Kevin Jang, Abdala Abdal-hay, Sašo Ivanovski, Sandleen Feroz

**Affiliations:** 1 School of Dentistry, Oral Health Centre Herston, The University of Queensland, 288 Herston Road, Herston, QLD 4006, Australias.ivanovski@uq.edu.au (S.I.); 2 Centre for Orofacial Regeneration, Rehabilitation and Reconstruction (COR3), School of Dentistry, The University of Queensland, Brisbane, QLD 4006, Australia

**Keywords:** dental erosion, biomimetics, enamel regeneration, dentine regeneration, remineralization

## Abstract

Dental erosion has emerged as a significant modern oral health problem, characterized by the chemical dissolution of tooth structure resulting from frequent exposure to intrinsic or extrinsic acids. With a high global prevalence ranging from 30% to 50% in children and 20% to 40% in adults, its management is a clinical priority to prevent long-term complications like dentine hypersensitivity and functional impairment. This review outlines the multifactorial etiology of erosion, encompassing dietary acids, gastroesophageal reflux, and reduced salivary flow. The historical context of oral care is explored, leading to a discussion on contemporary management strategies centered on remineralization. Fluoride ions play a crucial role by inhibiting demineralization, facilitating the formation of acid-resistant fluorapatite, and exerting antibacterial effects. A major focus is placed on advanced biomimetic, calcium phosphate-based topical agents such as Casein Phosphopeptide–Amorphous Calcium Phosphate (CPP-ACP), functionalized Tricalcium Phosphate (fTCP), and Hydroxyapatite (HAP), which effectively replenish lost minerals. The review further explores innovative methods, such as laser-assisted and electrically enhanced remineralization. Finally, it outlines next-generation regenerative strategies, including self-assembling peptides (P11-4), stem cell therapies, 3D bioprinting, and gene-editing (CRISPR) technologies, which aim to biologically regenerate lost enamel and dentine. The field is rapidly evolving from a preventive to a restorative paradigm, with future directions focusing on biologically based, minimally invasive therapies to fully restore tooth structure and function.

## 1. Introduction

Modern dentistry is increasingly guided by the paradigm of prevention, early diagnosis, minimal intervention, and the use of topical remineralizing agents to reduce the need for invasive restorative procedures [1]. Among oral health challenges, dental caries and its complications remain highly prevalent worldwide, while dental erosion has recently emerged as a significant “modern world problem” [1,2]. Although dental erosion and dental caries differ in their etiological and biochemical mechanisms, demineralized tooth surfaces caused by erosion are more prone to carious lesions and additional structural damage [1,3,4].

Recent data have clearly shown that dental erosion is prevalent not only in adults but also in children and adolescents across both developed and developing countries [1,5]. The accurate global prevalence of dental erosion remains unclear. However, recent meta-analyses and large-scale epidemiological studies indicate that the global prevalence of erosive tooth wear in children ranges from 30% to 50%, with the primary dentition specifically estimated at 35.6% (95% CI: 24.8–48.1) [6,7]. The reported prevalence of erosive tooth wear in permanent dentition is about 20–40%, but it may vary across specific cohorts [8].

According to the National Health and Medical Research Council (NHMRC), the prevalence of dental erosion in Australia among children ranges from 30% to 70%, with the primary dentition most affected [9]. In adults, nationally representative oral health trackers and surveys indicate that approximately 23–32% present with untreated dental disease, with specific studies reporting a dental erosion prevalence of around 23% [10]. These findings clearly indicate the significance of early intervention for the management of this condition, which, if left untreated, may lead to long-term complications such as dentine hypersensitivity, functional impairment, and compromised aesthetics [1]. Thus, it underscores the urgency of public health interventions that prioritize prevention, early recognition, and education for both children and adults.

Dental erosion refers to the chemical loss of mineralised tooth substance resulting from exposure to acids not produced by oral bacteria. This process involves the irreversible dissolution of enamel and dentine due to non-bacterial acid contact, including intrinsic (e.g., gastric acids) and extrinsic (e.g., dietary acids) sources [11]. The initiation and progression of dental erosion are multifactorial phenomena in which biological, chemical, and behavioural factors play crucial roles [1,5].

Intrinsic factors primarily include gastric acid exposure associated with gastroesophageal reflux disease (GERD), chronic vomiting, and eating disorders, which repeatedly subject tooth surfaces to highly acidic conditions [1,12]. However, the relationship between GERD and dental erosion remains inconclusive [13]. Although the World Congress of Gastroenterology reported a higher prevalence of dental erosion among patients with GERD, subsequent studies, particularly in pediatric populations, have yielded inconsistent findings [14,15]. The systematic reviews have also reported conflicting findings, largely due to methodological heterogeneity and flawed study designs. Despite recently published well-designed studies, evidence remains conflicting, underscoring the need for a comprehensive systematic review and meta-analysis to elucidate the role of GERD as a potential risk factor for dental erosion in children [16,17].

Extrinsic factors include dietary, occupational, and lifestyle-related sources of acid exposure. Frequent consumption of acidic beverages such as soft drinks, fruit juices, and sports drinks has been consistently associated with increased prevalence and the severity of dental erosion [18]. Jarvinen et al. were among the first to report a strong association between the frequency of consumption of acidic beverages and the severity of dental erosion [12], a finding subsequently supported by multiple studies [19,20,21,22,23]. Additional extrinsic risk factors include occupational exposure in battery and galvanizing factories, professional activities such as wine tasting and swimming, reduced salivary flow, high intake of acidic medications (e.g., aspirin), and frequent use of vitamin C–containing chewable tablets or gums [1,18].

Several studies have investigated the specific tooth surfaces affected by dental erosion [22,24,25,26,27]. Both lingual and buccal surfaces are susceptible to acidic challenges of intrinsic or extrinsic origin, leading to enamel demineralization [24]. Anatomical factors, including the movements of the tongue, lips, and cheeks, also play a crucial role in the localization and severity of erosive lesions [25].

Several classifications have been used in the literature to measure dental erosion [28,29]. Most indices developed for evaluating tooth surface loss in adults are not suitable for children. Similarly, the number of indices has been proposed, but no index fulfilling all relevant criteria has been found [28]. The Smith and Knight Tooth Wear Index is mostly used to measure all types of tooth wear in adults, including attrition, abrasion, and erosion [30]. O’Sullivan et al. used a modified form of this index in their studies and measured the erosion in children with gastroesophageal reflux [31]. Aine et al. proposed a more simplified classification of dental erosion in children with gastroesophageal disease. Each tooth was scored on a scale of 0–3. This classification can be used for primary, mixed, and permanent dentition [9,32].

Clinical diagnosis of dental erosion relies primarily on careful visual–tactile examination and recognition of characteristic lesion features [33]. These clinical criteria, often operationalised through indices and other tooth wear scoring systems, enable the grading of lesion severity for both individual patients and population-based studies. However, the clinical assessment of dental erosion remains challenging and limited [34]. Early erosive changes can be difficult to distinguish from physiological wear, photographic documentation and indices show only moderate inter- and intra-examiner reliability, and existing scoring systems were largely developed for epidemiological use rather than for monitoring lesion progression in individual patients [22,35]. In addition, there is no widely available chairside device that can reliably quantify small changes in mineral loss over time, which complicates both longitudinal clinical management and research on preventive or remineralizing interventions [36].

Historically, dental erosion was uncommon and mostly affected people with certain occupational or medical exposures to acid. Its prevalence increased markedly during the late 19th and 20th centuries, coinciding with industrial food processing and the widespread availability of carbonated beverages and acidic fruit-based drinks [37]. These dietary transitions introduced frequent extrinsic acid challenges to the oral environment, fundamentally altering the epidemiology of tooth surface loss and contributing to the recognition of dental erosion as a modern lifestyle-related condition [2,38,39,40].

Unlike previous reviews that primarily focus on epidemiology, risk factors, and conventional preventive measures, this review emphasizes the current understanding of dental erosion mechanisms, the protective role of saliva, and contemporary remineralization strategies, including peptide-guided mineralization, stem-cell-based therapies, bioengineered scaffolds, and gene-editing approaches. Additionally, the review critically addresses translational and regulatory challenges around clinical implementation, thereby providing a forward-looking framework that sets it apart in guiding future therapeutic development.

## 2. Role of Saliva and Fluoride-Based Topical Agents for Nonrestorative Management of Dental Erosion

Saliva plays a crucial protective role in preventing dental erosion by diluting, buffering, and clearing acidic ions [41,42,43,44,45,46,47,48,49,50,51,52,53,54]. This protective effect has been recognized for over a century, with Head (1912) first describing the buffering and neutralizing properties of saliva on acids [55,56]. Multiple studies have since highlighted a close association between dental erosion and salivary flow rate [20,53,57].

Clinical and experimental studies consistently demonstrate a close association between low salivary flow rate and an increased prevalence and severity of dental erosion [58,59]. When salivary flow is reduced dehydration or pathological conditions, acid clearance is reduced, and the oral environment remains undersaturated with respect to tooth mineral for longer periods, thereby accelerating erosive tissue loss from both intrinsic (e.g., reflux, vomiting) and extrinsic (dietary) acid sources [59].

Furthermore, saliva also contributes to the formation of an acquired pellicle composed of adsorbed proteins and other macromolecules, which can reduce the rate of enamel softening and surface dissolution during acid exposure [59,60].

Furthermore, the interaction between diet and enamel surface integrity has been explored extensively, with numerous in vitro and in vivo studies showing that frequent consumption of acidic beverages and foods substantially increases the risk and severity of erosive tooth wear [5,61,62].

It is also worth mentioning that, under favourable conditions, saliva alone can partially remineralize early erosive enamel lesions by depositing calcium phosphate phases, helping to restore surface hardness [63]. This concept has been further validated by numerous in vitro and in vivo studies, evaluating calcium- and fluoride-based products, and other remineralizing systems for their ability to enhance natural saliva-mediated repair and reduce mineral loss [1]. Akküç et al. investigated the remineralizing efficacy of three products, CPP-ACP, sodium fluoride, and herbal ingredients, against erosion [4].

Their findings indicate no significant difference between these products in increases in mineral density or reductions in lesion depth, surface area, and volume, thus supporting that several remineralizing agents are similarly effective in managing erosion-induced lesions. Overall, these agents can substantially enhance enamel surface remineralization and slow lesion progression; however, they do not provide complete, long-term protection against frequent or severe acidic challenges [4].

### 2.1. Role of Fluoride Ions in Inhibiting Demineralization

**Figure 1:**
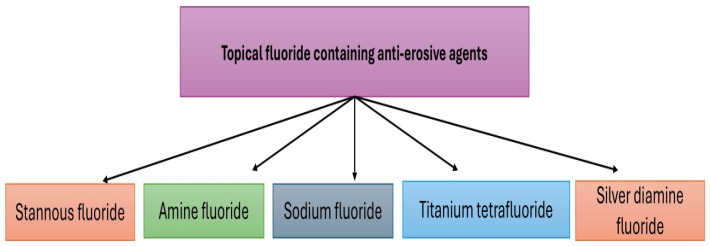
Commonly used fluoride-containing anti-erosive agents.

Topical application of fluoride for caries prevention has been known since 1980 [64]. Several in vitro studies have been carried out to evaluate the effects of fluoride-containing varnish and dentifrice on eroded enamel surfaces [65,66]. Similarly, the presence of fluoride in oral care products plays an important role in protecting the tooth surfaces against repeated erosive challenges [67,68]. Commonly used topical fluoride-containing agents are shown in Figure 1.

**Figure 2:**
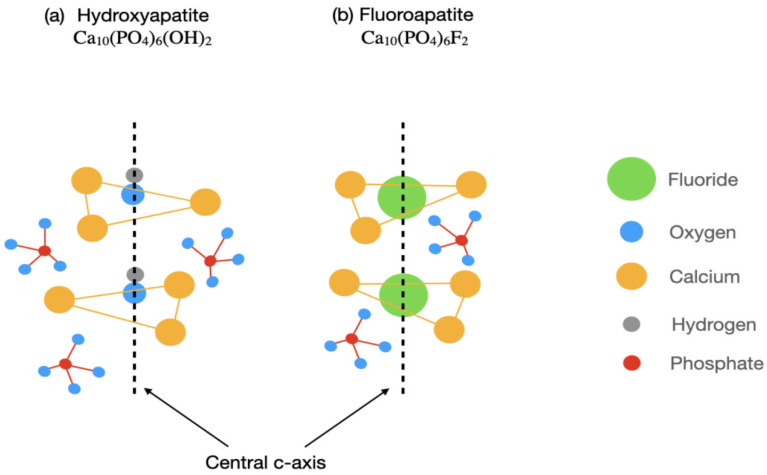
Schematic comparison of hydroxyapatite and fluoroapatite crystal structures. (**a**) Hydroxyapatite, Ca_10_(PO_4_) _6_(OH)_2_, showing hydroxyl (OH^−^) ions aligned along the central c-axis. (**b**) Fluoroapatite, Ca_10_(PO_4_) _6_F_2_, illustrating substitution of hydroxyl groups by fluoride (F^−^) ions along the same axis. Calcium (yellow), phosphate groups (red), oxygen (blue), hydrogen (grey), and fluoride (green) are indicated. The dashed line denotes the crystallographic c-axis, highlighting the structural stabilization associated with fluoride incorporation [74].

Mazzoleni et al. conducted an interesting in vitro analysis to evaluate the protective effect of three different alloplastic materials against erosive wear [67]. It was reported that the fluoride-containing varnishes and toothpastes demonstrated superior remineralizing and protection against dental erosion compared to non-fluoride toothpaste. Thus, the availability of fluoride ions in the oral cavity also promotes remineralization by enhancing crystallization kinetics. This results in reduced demineralization by significantly lowering the solubility of the apatite phase. Figure 2 shows a basic hydroxyapatite (HA) crystal structure to illustrate the role of fluoride ions in remineralization [69]. Ionic substitutions in the HA crystal structure, when combined with external ions, modify its properties. Cationic substitutions involve the replacement of hydroxyl ions with either larger halide (F¯, Cl¯) ions or the replacement of phosphate functional groups by silicate or carbonate groups. It has been observed that the application of certain fluoride-containing topical agents can inhibit the matrix metalloproteinases (MMPs) in dentine matrix [70,71]. The modification of salivary pellicle to increase acid resistance has also been reported previously [72,73].

The application of 2000 ppm sodium fluoride solution to the eroded dentin surfaces prior to brushing has shown to significantly reduce further abrasion, supporting the protective role of topical fluoride against mechanical wear following acid challenges [75]. Several studies have been reported to understand the mechanism of tooth demineralization prevention by the application of topical fluoride-containing products.

### 2.2. Mechanisms of Fluoride in Demineralization Inhibition

In general, four mechanisms are involved in minimizing tooth demineralization.

**Surface Protection via Plaque Fluid Fluoride**: Fluoride ions in plaque fluid penetrate into the tooth subsurface and protect apatite crystals from acidic dissolution at low pH. Systematically incorporated fluoride ions are not sufficient to inhibit the effects of these acidic ions [76].

**Facilitation of Remineralization:** When the oral pH returns to 5.5 or above, fluoride accelerates remineralization by attracting calcium and phosphate ions, forming a new mineral phase, fluorapatite. This remineralized surface exhibits increased resistance to subsequent acid attacks [76,77].

**Antibacterial Action by Intracellular Fluoride**: Under acidic conditions, fluoride ions combine with hydrogen to form HF, which readily penetrates bacterial cell walls. Inside the cell, HF dissociates, releasing fluoride ions that disrupt bacterial metabolic enzymes and reduce acid production [78].

**Reservoir Effect for Prolonged Protection:** After topical application (e.g., toothpastes, gels, varnishes), fluoride is retained in intraoral reservoirs (like saliva or oral mucosa) and slowly released over time, maintaining a protective effect even between exposures [78,79].

Based on the general mechanisms outlined above, it is crucial to understand that different fluoride formulations exhibit distinct properties influencing their clinical performance. These differences arise from the nature of the accompanying cation, the formulation’s pH, and interactions with enamel, dentine, and the salivary pellicle [80].

- **Stannous fluoride (SnF_2_):**

Stannous fluoride combines fluoride ions with divalent tin, which can precipitate on enamel and dentine surfaces as stannous-rich deposits, forming a relatively acid-resistant surface layer and partially occluding dentinal tubules [71,81]. This results in effective protection against erosive challenges. Since stannous fluoride was introduced in the mid-20th century, its broader use in daily oral care has been enabled by “stabilized” SnF_2_ formulations developed from the 1990s onwards, which incorporate chelating agents such as gluconate to maintain tin in solution and increase its bioavailability. In these systems, the chelator helps prevent premature reaction of SnF_2_ with abrasive or surfactant components in toothpastes, thereby preserving the ability of stannous ions to form protective surface complexes and to deliver a broad spectrum of benefits, including erosion/abrasion control, dentine hypersensitivity reduction, and antibacterial activity [82].

One potential limitation of SnF_2_ dentifrices is their potential to cause extrinsic tooth discoloration. This drawback has been partially addressed by incorporating calculus- and stain-controlling polyphosphates, such as sodium hexametaphosphate (SHMP), into stabilized SnF_2_ formulations [82].

- **Amine fluoride**:

Amine fluorides are organic, surfactant-based fluoride compounds that exhibit strong adsorption to enamel, dentine, and oral soft tissues, thereby enhancing fluoride retention at the tooth surface and within the acquired pellicle [83]. Owing to their amphiphilic molecular structure, amine fluorides facilitate the formation of a more homogeneous, fluoride-rich surface film, which has been shown to improve resistance to subsequent acid challenges [80]. Nevertheless, their clinical application for treating erosive tooth wear remains limited by the lack of long-term, high-quality clinical evidence. Most available data are derived from in vitro studies, with substantially stronger support currently existing for their efficacy in caries prevention.

- **Sodium fluoride (NaF)**:

Sodium fluoride is the most widely used inorganic fluoride source in toothpastes, mouthrinses, and varnishes [83]. NaF primarily promotes the formation of a calcium fluoride-like surface reservoir and enhances fluorapatite formation during remineralization, reducing mineral loss under repeated acid challenges.

However, at typical toothpaste concentrations and neutral pH, NaF forms only a thin surface reservoir, that may be insufficient to protect the tooth surface against conditions that result in repeated or prolonged acid exposure [83,84,85].

- **Titanium tetrafluoride (TiF_4_):**

The preventive role of topically applied fluorides is well established. However, titanium tetrafluoride (TiF_4_) has demonstrated superior protective effects against both dental caries and erosive tooth wear compared with conventional fluorides such as sodium fluoride (NaF), stannous fluoride (SnF_2_), and acidulated phosphate fluoride (APF) [86,87]. The unique interaction of TiF_4_ with dental hard tissues results in the formation of a tenacious, glaze-like, titanium-rich surface layer that enhances resistance to acid dissolution [88]. In addition, TiF_4_ exhibits rapid and extensive fluoride uptake in enamel, dentine, and root surfaces, an effect attributed to the synergistic action of titanium ions with fluoride. This chemically modified surface layer differs from the calcium fluoride–like precipitates typically formed by conventional fluorides and is considered more stable under acidic conditions, thereby offering prolonged protection against erosive challenges [89].

Although topical fluorides can reduce erosion by promoting the deposition of CaF_2_-like reservoirs on softened enamel surfaces, these precipitates dissolve readily under acidic conditions, limiting their long-term protective efficacy [87].

Enamel surfaces treated with TiF_4_ form a thick, acid-resistant coating that remains stable even after prolonged exposure to strong acids, thereby significantly reducing mineral loss and surface softening [88]. Similarly, comparative in vitro studies have demonstrated that TiF_4_ exhibits superior efficacy to NaF and SnF_2_ in reducing erosive lesion depth and maintaining enamel surface integrity, with treated specimens frequently showing inhibition of erosive lesion development [90,91,92]. These findings highlight the potential of TiF_4_ as a promising agent for erosion prevention, although its clinical application remains constrained by limited long-term clinical evidence and concerns about its low pH and mucosal tolerability [89,93].

- **Silver diamine fluoride (SDF):**

Silver diamine fluoride (SDF) is a polyvalent fluoride compound containing metal cations and has been proposed as a potential agent for preventing dental erosion [94,95]. The World Health Organization has listed SDF as an essential medicine, recognizing its safety and efficacy in the management of oral diseases [96]. The widely used 38% SDF formulation contains approximately 44,800 ppm fluoride and 253,900 ppm silver ions [96,97]. Experimental studies indicate that SDF promotes the formation of a protective surface barrier composed of calcium fluoride–like deposits (CaF_2_), silver chloride (AgCl), and metallic silver on dental hard tissues [72,94]. In addition, SDF can react with hydroxyapatite to form fluorapatite crystals, thereby increasing enamel microhardness [94]. Its inhibitory effect on matrix metalloproteinases further contributes to preservation of the demineralized organic matrix, potentially delaying the progression of dentinal erosion [96].

Despite these promising mechanisms, evidence supporting the anti-erosive efficacy of SDF remains limited. To date, only a small number of laboratory studies have evaluated its protective effects against dental erosion [95,98]. Ainoosah et al. demonstrated that SDF significantly reduced erosive damage in bovine enamel [72], whereas Cunha et al. reported that 10% SDF inhibited phosphorus release associated with erosion in human primary enamel [98]. Additionally, Suresh et al. observed that 38% SDF reduced enamel microhardness loss in primary teeth exposed to pediatric liquid medications [99]. The greater susceptibility of primary enamel to erosion, attributable to its lower mineral content and reduced thickness compared with permanent enamel, may further accentuate the potential clinical relevance of SDF in paediatric populations [99].

## 3. Calcium Phosphate-Based Topical Agents

Modern management of dental erosion emphasizes not only the prevention of further demineralization but also the active promotion of remineralization using biomimetic agents [100,101].

Calcium phosphate-based systems are particularly important because their composition closely resembles the inorganic mineral phase of enamel and dentine, thereby supporting the redeposition of minerals lost during erosive challenges [102]. When demineralization occurs, the availability of calcium and phosphate ions can favour mineral reprecipitation, helping to arrest and partially reverse early erosive lesions. However, in individuals with reduced salivary flow or frequent acid exposure, endogenous saliva alone is often insufficient to maintain this supersaturated state, making it clinically relevant [80,103].

Several studies have demonstrated the effectiveness of calcium phosphate-based agents when applied topically to replenish minerals lost due to repetitive acidic challenges from erosion [104]. Calcium phosphate-based agents to inhibit erosion include casein phosphopeptides, hydroxyapatite (HAP) based products, β-tricalcium phosphate, calcium silicate sodium phosphate, pyrophosphate, calcium lactate, Sodium hexametaphosphate (SHP)/Sodium trimetaphosphate (TMP), and linear calcium phosphate [95]. Collectively, these materials increase the availability of calcium and phosphate at the tooth surface, reduce enamel softening and slow the progression of erosion, particularly when combined with appropriate fluoride exposure and dietary acid control.

### 3.1. Casein Phosphopeptide Amorphous Calcium Phosphate (CPP-ACP)

CPP-ACP complex, patented by the University of Melbourne, Australia, received recognition in 1999 when the U.S. Food and Drug Administration (FDA) approved the use of Recaldent in chewing gum formulations at concentrations of up to 5% *w*/*w* [105].

CPP-ACP is a bioactive compound derived from casein, a milk protein, and has been widely studied for its potential to remineralise dental tissues. CPP stabilises calcium and phosphate ions in an amorphous state, forming nanoclusters that can localise at the tooth surface. These clusters bind to dental plaque and enamel, maintaining a supersaturated state of calcium and phosphate ions around the enamel surface, which helps inhibit demineralisation and promotes remineralisation [1].

Reynolds and colleagues demonstrated that CPP-ACP exhibits a strong affinity for both tooth surfaces and plaque bacteria, thereby localizing high concentrations of amorphous calcium phosphate (ACP) in close proximity to tooth surfaces. When the pH of the oral cavity decreases, this CPP-ACP buffers free calcium and phosphate ions, maintaining a state of supersaturation. This dynamic not only promotes remineralization but also effectively inhibits the demineralization processes on enamel surfaces [105,106]. Several in vitro and in vivo studies have demonstrated that CPP-ACP can significantly reduce the erosive potential of acidic challenges by forming a protective layer over the enamel and by facilitating the repair of subsurface lesions [3,4]. Additionally, it has been shown to enhance enamel resistance to future acid attacks, particularly when incorporated into dental products such as chewing gums, topical creams (e.g., GC Tooth Mousse), and varnishes [5]. CPP-ACP demonstrates consistent short-term benefits in reducing enamel surface loss and increasing microhardness in erosive and early carious lesions [107]. Randomised controlled trials (RCTs) generally report modest but statistically significant improvements compared with placebo or no-treatment controls [108,109].

Overall, CPP-ACP is considered a promising adjunctive agent in the preventive and therapeutic management of dental erosion due to its remineralising and protective properties.

### 3.2. Tricalcium Phosphate (TCP)

Tricalcium phosphate (TCP), in its functionalised forms, has gained attention as a remineralising agent capable of counteracting erosion by replenishing calcium and phosphate ions necessary for enamel repair. TCP is a bioactive calcium phosphate compound that mimics the mineral phase of tooth enamel and dentin. When used in oral care products, it provides a reservoir of bioavailable calcium and phosphate ions, which are essential for enamel remineralisation [23]. However, the effectiveness of native TCP is limited by its tendency to prematurely react with fluoride or other agents, thereby reducing ion availability. To overcome this, functionalised TCP (fTCP), often coated with organic agents such as fumaric acid, is used to stabilise and regulate ion release in the oral environment [22].

Recent in vitro studies have demonstrated that fTCP, when combined with fluoride, significantly enhances the remineralisation of acid-softened enamel compared with fluoride alone. The synergistic effect is attributed to the controlled release of calcium and phosphate ions at the tooth surface, which promotes the formation of fluorapatite—a mineral more resistant to acid dissolution than hydroxyapatite [12].

Clinical research supports the preventive role of TCP in high-risk individuals, including those with low salivary flow or high dietary acid exposure. Studies using fTCP-containing toothpaste showed reduced enamel wear under erosive challenges, suggesting a protective effect by forming a mineral-rich layer that resists acid attack [15]. Additionally, TCP formulations are biocompatible and low-toxic, making them suitable for long-term use. In head-to-head trials, fTCP–fluoride formulations tend to outperform fluoride alone but do not consistently exceed the performance of CPP-ACP–fluoride combinations [110]. The principal advantage of fTCP lies in controlled delivery and compatibility with fluoride, rather than in independent remineralizing capacity [111].

Despite promising results, variability in study designs and formulations calls for more standardised clinical trials to determine optimal concentrations and combinations of TCP with fluoride or other agents. As research progresses, TCP is poised to become a key component in preventive dentistry for managing early stages of dental erosion and promoting hard tissue regeneration.

### 3.3. Hydroxyapatite-Based Products

Hydroxyapatite (HAP), a biomimetic form of calcium phosphate that closely resembles the natural mineral composition of tooth enamel, has emerged as a promising agent for both preventing and repairing erosive damage.

HAP exhibits bioactivity and biocompatibility, enabling it to integrate with natural tooth surfaces. When applied topically, nano-hydroxyapatite particles can penetrate demineralised enamel and bind to exposed hydroxyapatite crystals, promoting the regeneration of lost mineral content [19]. Unlike fluoride, which primarily strengthens enamel by forming fluorapatite, HAP physically fills micropores and surface defects, acting as a scaffold for remineralisation [20].

Recent in vitro and in situ studies have demonstrated that HAP-containing toothpaste and mouthrinses significantly reduce the progression of enamel erosion by forming a protective layer on the tooth surface [21]. This layer not only shields against acidic challenges but also contributes to a smoother surface morphology, reducing plaque retention and bacterial adhesion. Furthermore, HAP is suitable for individuals seeking fluoride-free alternatives, such as children, pregnant women, or those with fluorosis concerns [11].

Evidence also supports the use of HAP in combination with other bioactive agents. For example, formulations combining HAP with xylitol or arginine have shown improved remineralisation potential and anti-caries efficacy [10]. HAP’s application extends to both primary and permanent teeth, making it a versatile option in preventive and restorative dentistry.

Consequently, HAP appears particularly beneficial for patients seeking fluoride-free options, but not clearly superior in high-risk erosive conditions. Despite its promising performance, more long-term, standardised clinical trials are needed to compare its efficacy with conventional fluoride treatments under various erosive conditions [112]. However, current findings indicate that HAP can play a significant role in the non-invasive management of dental erosion by mimicking natural enamel repair mechanisms.

### 3.4. Calcium Silicate and Sodium Phosphate (CSSP)

Preventing or reversing the early stages of erosion is critical to preserving dental hard tissues [1]. Recently, biomimetic mineralising systems, such as calcium silicate and sodium phosphate (CCSP), have been studied for their potential to enhance enamel resistance and facilitate remineralisation [113].

Calcium silicate is a bioactive compound that, when exposed to aqueous environments, releases calcium ions that can form hydroxyapatite-like mineral layers on the enamel surface [24]. Sodium phosphate complements this process by supplying phosphate ions, essential for hydroxyapatite crystal growth and enamel remineralisation. Together, CCSP provides a dual source of mineral precursors that not only promote remineralisation but also form a protective barrier against acid challenges [25].

The mechanism of action is two-fold: CCSP facilitates nucleation of calcium phosphate phases directly on the enamel surface and also interacts with salivary ions to enhance remineralisation kinetics. This makes it effective under acidic and erosive conditions, where mineral loss is accelerated. Several in vitro and in situ studies have shown that CCSP can restore enamel surface microhardness, reduce mineral loss, and increase resistance to future erosion [26].

Compared with fluoride-only treatments, CCSP offers the advantage of forming a physical mineral layer on the tooth surface, that can act as an immediate barrier. Additionally, CCSP is fluoride-compatible and can be included in dual-action formulations to maximise remineralisation outcomes [27].

Clinical research indicates that CCSP-containing pastes and rinses are well tolerated and effective in reducing sensitivity, improving surface hardness, and enhancing enamel protection over extended periods [28]. Their application is suitable for both preventive and restorative protocols, especially in patients with high acid exposure or those unable to tolerate fluoride.

However, while the preliminary data are promising, long-term clinical trials with standardised methodologies are still limited. Future research should explore optimal application frequency, synergistic effects with fluoride, and long-term outcomes in diverse populations.

### 3.5. Other Calcium Phosphate Based Remineralizing Agents

Modern management of dental erosion emphasizes not only the prevention of further demineralization but also the promotion of remineralization using bioavailable minerals. Calcium phosphate-based remineralizing agents play a pivotal role in restoring mineral content and improving resistance to acidic challenges. Calcium and phosphate are essential for tooth mineralization, and their availability in saliva is critical in natural remineralization. When demineralization occurs, the availability of these ions can help reverse early erosive lesions. However, in patients with insufficient saliva or high acid exposure, exogenous supplementation through remineralizing agents becomes necessary.

#### 3.5.1. Calcium Lactate

Calcium lactate, a calcium salt of lactic acid, is a bioavailable calcium source. It dissociates easily in saliva, releasing calcium ions that participate in remineralization. Studies have shown that calcium lactate, when applied topically or incorporated into toothpaste or mouth rinses, enhances enamel microhardness and reduces surface roughness after erosive attacks [14]. It has also been used in combination with fluoride to potentiate remineralizing effects.

#### 3.5.2. Pyrophosphate

Pyrophosphates, particularly tetrasodium pyrophosphate, have dual functionality. They prevent the formation of calculus by binding calcium ions, thereby reducing mineral precipitation. However, at controlled concentrations, they contribute to remineralization by supplying phosphate ions that can integrate into hydroxyapatite crystals. Pyrophosphates have been shown to reduce enamel erosion in vitro when used in conjunction with calcium and fluoride [15].

#### 3.5.3. Linear Sodium Phosphate

Linear sodium phosphate is a water-soluble source of inorganic phosphate. It assists in remineralization by maintaining phosphate ion concentrations in the oral environment, favoring supersaturation with respect to hydroxyapatite. Research indicates that linear phosphates, when used with calcium salts and fluoride, can effectively reduce mineral loss and promote the formation of fluorapatite, a more acid-resistant mineral [16].

#### 3.5.4. Sodium Hexametaphosphate (SHMP)

SHMP is a polyphosphate known for its chelating properties. It adheres to tooth surfaces, forming a protective film that enhances mineral retention. SHMP can also bind calcium ions and release them gradually, providing a sustained source of minerals. Its surface-binding ability reduces enamel solubility and enhances fluoride uptake [17].

#### 3.5.5. Sodium Trimetaphosphate (STMP)

STMP has emerged as a promising agent due to its ability to form stable complexes with calcium. It enhances fluoride efficacy and supports the precipitation of calcium phosphate phases. When incorporated into toothpaste formulations, STMP has shown significant protective effects against erosive wear, particularly when combined with low concentrations of fluoride [18].

The combined use of these agents leverages the synergistic effects of calcium and phosphate availability, increased fluoride retention, and reduced enamel solubility. Current research focuses on optimizing their concentrations and combinations to enhance efficacy and reduce erosion in both in vitro and in vivo models, as shown in Table 1.

## 4. Innovative Methods/Tools for Effective Application of Remineralizing Agents

Innovative advanced methods, when combined with biomimetic approaches for tooth surface remineralization, could pave the way for improved oral health outcomes. These methods combine physical treatment with chemical agents to enhance the natural remineralization process and provide alternative strategies for preventing and treating early stages of cavitated lesions [114].

Laser-Assisted Mineralization is among the most promising thermal treatment approaches for enamel remineralization. Laser technology leverages photothermal effects to create optimal conditions for crystal growth by heating the treatment area and transforming reaction conditions into a hydrothermal environment. This process accelerates the mineralization of dental enamel while controlling hydroxyapatite crystal formation in affected areas [114]. The carbon dioxide laser, when applied at an optimized wavelength, effectively aligns with the absorption peak of carbonated hydroxyapatite, resulting in significant inhibition of demineralization [115,116]. Additionally, femtosecond pulsed lasers can remineralize enamel by sintering artificial fluorapatite powder containing iron oxide nanoparticles, which act as thermal antennas to absorb laser photons and promote densification into adherent layers. However, the main limitation of diode laser treatment is the risk of overheating effects that may damage dentinal and pulpal nerve cells, thus necessitating further research to investigate alternative laser sources [116].

Rotary Evaporation and Electrical Enhancement techniques offer controlled approaches to crystal formation and accelerated remineralization. Rotary evaporation provides a simple yet effective method for regulating crystal formation and regenerating enamel-like structures on various substrates, producing highly ordered structures at controlled thickness much more rapidly than traditional hydrothermal approaches [117]. This technique utilizes silk fibroin as a crystal-formation modulator in remineralizing solutions containing calcium, phosphate, and fluoride, resulting in regenerated enamel-like crystals with microstructure and mechanical properties comparable to native enamel [118].

Electrically enhanced remineralization presents an innovative clinical approach that can rapidly remineralize dental cavities without the need of conventional filling and drilling procedures [119]. This painless procedure applies remineralizing agents to carious lesions, followed by a brief electric field application to accelerate mineral penetration into the lesion [119]. Furthermore, electrodeposition techniques, including both electrolytic deposition and electrophoretic deposition, can create uniform coating layers under electrical fields at relatively low temperatures with controlled crystallinity, even on porous or uneven surfaces. Although these methods showed promising results, their clinical translation remains limited due to the high electric-field conditions required for the procedures [120].

## 5. Next Generation Strategies to Repair and Regenerate Enamel and Dentine

Recent advancements in regenerative dentistry offer significant potential to address the key limitations of the conventional calcium and fluoride-based topical therapies discussed above. These strategies have revolutionized research on the restoration and regeneration of lost dental tissues, including enamel, dentine, pulp, and periodontal structures. These innovative approaches focus on the biological restoration of lost dental hard tissues through biomimetic processes, advanced biomaterials, and cutting-edge cellular and molecular technologies.

### 5.1. Advanced Biomimetic Enamel Regeneration

#### 5.1.1. Self-Assembling Peptide Systems

P11-4 technology represents a significant development in biomimetic strategies for enamel repair, with particular relevance to erosive lesions [121]. This rationally designed 11-amino acid peptide demonstrates a remarkable ability to self-assemble into three-dimensional scaffolds that mimic the natural enamel matrix. The peptide diffuses into erosive lesions and forms biomimetic nucleation sites for hydroxyapatite crystal formation, effectively restoring the hierarchical structure of damaged enamel. Recent studies demonstrate that P11-4 treatment can achieve superior remineralization outcomes compared to conventional approaches, particularly when applied to acid-softened enamel surfaces characteristic of erosive wear [122].

Biomimetic liquid-based systems represent an emerging class of peptide-guided enamel regeneration strategies [123]. In this approach, short amelogenin-derived peptides such as P26 are combined with stabilized calcium phosphate clusters to ensure ordered crystal growth on acid-etched enamel. In vitro studies have shown that these peptide–mineral assemblies can infiltrate demineralized enamel, where the calcium phosphate clusters undergo controlled crystallization to form continuous enamel-like hydroxyapatite layers, improving surface hardness and acid resistance compared with untreated, eroded controls [123,124,125]. Furthermore, the recent studies reported from Zhejiang University using calcium phosphate ion clusters have demonstrated that micrometre-scale enamel-like layers (approximately 2–3 µm) can be regenerated on etched enamel surfaces within about 48 h under experimental conditions, illustrating the potential for relatively rapid biomimetic repair [125]. The reported findings are limited to laboratory models, and clinical protocols for treating erosive tooth wear have not yet been established.

Biomimetic liquid regeneration remains at a preclinical stage of development; however, initial findings indicate considerable potential for managing erosive enamel loss, as it aims to replace mineralized tissue lost to acid dissolution rather than merely enhancing conventional remineralization processes.

#### 5.1.2. Epitaxial Remineralization Techniques

Advanced epitaxial remineralization strategies employing calcium phosphate ion clusters (CPICs) have emerged as promising approaches for repairing tooth enamel [126,127]. The presence of stabilized nanoclusters of calcium phosphate acts as a ‘mineralization frontier’, promoting epitaxial growth of enamel-like hydroxyapatite that is continuous with the underlying prism structure on demineralized surfaces. The in vitro studies have reported that the regeneration of enamel-like layers on etched enamel significantly increases surface hardness [104,128]. However, when compared to sound enamel surfaces, these regenerated layers remain thinner and exhibit lower mechanical properties. Thus, these technologies will open new avenues for incorporating minimally invasive methods to repair lost enamel, either partially or completely, due to acid dissolution

### 5.2. Regenerative Dentine Repair Strategies

#### 5.2.1. Biomimetic Dentin Remineralization

Bottom-up remineralization strategies are based on the use of biomimetic analogues of non-collagenous dentine matrix proteins to regulate apatite formation within demineralized collagen [129,130]. These systems are usually based on amorphous calcium phosphate (ACP) nano-precursors stabilized by polyanionic analogues of dentine matrix proteins, which facilitate both intrafibrillar and interfibrillar nucleation of hydroxyapatite within the gap zones of collagen fibrils [130]. Through this mechanism, the mechanical properties of demineralized dentine can be partially restored while the collagen matrix is protected from further acid- and enzyme-mediated degradation. These approaches hold a great potential for the restorative treatment of erosive tooth wear, where exposed dentine is characterized by substantial mineral depletion, as they aim to regenerate the existing collagen scaffold with newly formed apatite rather than simply occluding the dentine surface [131].

More recently, amelogenin-derived peptide therapies have demonstrated considerable potential for dentine repair under demineralizing conditions. The mineralization-directing peptide sADP5, derived from amelogenin, promotes a layer-by-layer, peptide-guided remineralization process that results in the formation of an infiltrating mineral layer together with peritubular mineralization on exposed human dentine, leading to durable tubule occlusion [130]. In vitro studies have shown that the newly formed mineral exhibits a calcium phosphate composition consistent with hydroxyapatite and demonstrates hardness and reduced elastic modulus values significantly greater than those of demineralized dentine and, in some reports, comparable to or exceeding those of sound dentine [132]. Importantly, these mineralized layers remain structurally integrated following thermal aging. Collectively, these findings suggest that amelogenin-derived peptides represent a promising biomimetic strategy for reinforcing erosively exposed dentine and enhancing its resistance to mechanical and thermal challenges in the oral environment. However, there is a dire need for long-term clinical trials to translate these in vitro findings into predictable clinical outcomes.

#### 5.2.2. Stem Cell-Based Regenerative Approaches

Dental pulp stem cell-based approaches offer a promising option for treating advanced erosive lesions compromising the dentin–pulp complex [133]. Several dental stem cell populations, including dental pulp stem cells (DPSCs), stem cells from human exfoliated deciduous teeth (SHEDs), and stem cells from the apical papilla (SCAPs), have demonstrated substantial regenerative capacity [133,134]. The results of in vivo studies and early clinical investigations indicate that these cells can generate dentin- and pulp-like tissues and contribute to the regeneration of the surrounding supportive structures [134,135].

Building on these advancements, recent pre-clinical trials have been initiated to further explore genetic engineering strategies for the regenerative potential of dental stem cells [135,136]. In particular, CRISPR-modified DPSCs engineered to upregulate factors such as brain-derived neurotrophic factor (BDNF) have shown enhanced odontogenic differentiation and increased deposition of dentin-like tissue in experimental models [136,137]. While these findings demonstrate the long-term potential of gene-enhanced stem cell therapies for managing severe erosive damage, it is important to mention that they are still in the early experimental stages [137]. Major translational challenges include genetic stability, immunogenicity, durability of regenerated tissues, and control of off-target gene-editing effects. Furthermore, large-animal studies and long-term clinical data are still limited. These issues need to be addressed before progression to routine clinical application.

#### 5.2.3. Advanced Biomaterial Scaffolds

Hydroxyapatite–collagen scaffolds have emerged as versatile platforms for dental hard-tissue regeneration. These composites combined the biocompatibility and cell-adhesive properties of collagen with the bioactivity of calcium phosphate ceramics, providing a three-dimensional framework that supports cell attachment, proliferation, and matrix deposition. They can be fabricated by freeze-drying, electrospinning, or 3D bioprinting to generate highly porous architectures that facilitate cell penetration, neovascularization, and efficient nutrient and waste exchange [138,139,140].

Bioactive glass integration further enhances scaffold functionality by enabling controlled release of therapeutic ions such as calcium, phosphate, and silicate [140]. When bioactive glass is incorporated into collagen-based matrices, the resulting composites not only support mineralized tissue formation but also exhibit antimicrobial and osteo-inductive properties. These composite scaffolds are particularly promising for the reconstruction of severe erosive lesions, where substantial enamel and dentine loss requires the complete replacement of hard tissues rather than just sub-surface remineralization [141,142].

Customized scaffold fabrication through 3D bioprinting enables the creation of patient-specific regenerative constructs [143,144]. This technology allows spatially precise placement of cells, biomaterials, and signalling molecules to produce complex architectures that approximate the hierarchical organization of native dental tissues. In the context of erosive wear, bioprinting offers the potential to generate scaffolds tailored to the morphology of individual defects, thereby providing targeted regenerative support. Similarly, bioink development has progressed to include formulations containing living cells, growth factors, and bioactive inorganic components. These bioinks can be printed into constructs capable of supporting both hard and soft tissue regeneration, which can be employed for the repairment of advanced erosive lesions that simultaneously affect enamel, dentine, and the underlying pulp–dentin complex [145].

#### 5.2.4. Growth Factor-Based Regenerative Strategies

Brain-derived neurotrophic factor (BDNF) has shown promising potential for enhancing dentin–pulp regeneration [146]. BDNF can stimulate proliferation and odontoblast-like differentiation of dental pulp stem cells and support neurovascular components within the pulp, thereby promoting reparative dentin formation and maintenance of pulp sensibility [146,147]. Although these studies have mainly been demonstrated in caries- or injury-related models, such neurotrophic factor-based approaches may, in the future, be adapted to advanced erosive lesions in which the dentin–pulp complex is compromised.

Bone morphogenetic proteins, particularly BMP-2 and BMP-7, are well-characterised growth factors with strong odontogenic and angiogenic activity [148,149]. When incorporated into suitable carriers or scaffold systems, they can provide sustained local release, enhancing the formation of dentin-like tissue and supporting regeneration of a vascularised pulp-like tissue. These properties make BMP-based delivery systems attractive candidates for biologically restoring deep, non-carious defects, including severe erosive lesions, although issues of dose control, ectopic mineralization, and long-term safety still limit clinical translation.

#### 5.2.5. Cell-Free Regenerative Technologies

Exosomes and other extracellular vesicles derived from dental stem cells offer a cell-free alternative to direct cell transplantation [150].

These nano-sized vesicles transport proteins, lipids, and nucleic acids that can modulate inflammation, stimulating angiogenesis, and promote matrix deposition, and have facilitated the pulp–dentin regeneration in pre-clinical trials when combined with biocompatible scaffolds [151,152]. When compared with cell-based therapies, extracellular vesicle approaches may provide advantages such as lower immunogenicity, no risk of uncontrolled cell proliferation, and easier storage and handling [153].

Engineered extracellular vesicles can be loaded with defined therapeutic cargoes, including growth factors, microRNAs, and other signalling molecules, to enhance or direct their regenerative potential [152,154]. Overall, these tailored vesicles could be designed to address the specific biological features of erosive lesions, for example, by promoting mineralisation of exposed dentin while modulating pulpal inflammation and neurovascular repair. At present, these strategies remain at an early experimental stage, but they represent a promising future avenue for minimally invasive, biologically based management of severe erosive tooth wear.

### 5.3. Clinical Translation and Future Directions

The clinical translation of regenerative strategies for erosive tooth wear aligns closely with contemporary principles of minimally invasive dentistry, which prioritise preservation of remaining tooth structure while supporting biological repair [1,155]. Conservative treatment protocols based on additive approaches aim to restore only tissue lost to erosive processes, thereby avoiding unnecessary removal of sound enamel or dentine. Within this framework, integrating regenerative technologies with adhesive restorative techniques offers a promising pathway to improve clinical outcomes in erosive wear management [100].

Early intervention is likely to play a critical role in the success of these approaches. The application of regenerative therapies at the initial stages of erosive damage, particularly during early dentine exposure, has the potential to limit lesion progression and preserve pulpal vitality [133]. For more advanced or complex erosive lesions, combination therapy approaches are increasingly being explored [155]. Multi-modal regenerative strategies that integrate stem cells, bioactive molecules, biomimetic materials, and gene-based modulation techniques may provide a more comprehensive solution for tissue repair [142]. The synergistic effects of combining biological and material-based approaches are particularly relevant in cases when both enamel and dentine are affected, and single-modality treatments may be insufficient. Similarly, the development of smart, responsive regenerative systems represents an emerging direction in this field [145]. Advances in nanotechnology have enabled the design of materials that respond dynamically to the oral environment, including fluctuations in pH, bacterial activity, and mechanical loading. Such systems offer the potential for site-specific, on-demand therapeutic responses tailored to the conditions characteristic of erosive tooth wear [128,130].

However, it is important to acknowledge that the majority of the findings from next-generation regenerative approaches, including peptide-guided mineralization systems, ion-cluster-based enamel regeneration, stem cell therapies, gene editing, and extracellular vesicle technologies, remain supported by in vitro and small-animal studies [156]. Translation of these technologies to routine clinical practice requires standardized manufacturing protocols, dose optimization, long-term safety evaluation, and randomized controlled clinical trials. Similarly, stem cell-based and gene-editing approaches are subjected to higher regulatory barriers.

Under current United States Food and Drug Administration (FDA) and European Medicines Agency (EMA) guidelines, these therapies are classified as advanced therapy medicinal products (ATMPs) or human cells, tissues, and cellular and tissue-based products (HCT/Ps), requiring rigorous evaluation of genetic stability and tumorigenic potential, immunological safety, manufacturing reproducibility under Good Manufacturing Practice (GMP) conditions, long-term clinical performance, and post-treatment monitoring for any delayed adverse effects [157]. These regulatory and ethical constraints substantially extend the development timelines and increase the translational complexity in dental applications.

Collectively, recent advances in regenerative approaches for erosive tooth wear signify a shift away from conventional invasive procedures toward biologically driven repair and regeneration. While many of these technologies remain under preclinical or early translational investigation, their continued development and clinical validation may ultimately transform the management of erosive tooth wear by providing sustainable, biologically integrated solutions that preserve natural tooth structure, vitality, and function.

## 6. Conclusions

Dental erosion is a growing global concern driven by dietary acids, intrinsic gastric factors, reduced salivary function, and behavioural habits, underscoring the need for early diagnosis and prevention. While conventional approaches such as fluoride, CPP-ACP, functionalized TCP, hydroxyapatite, and calcium silicate-based technologies remain effective in reducing mineral loss and enhancing enamel resistance, modern dentistry is rapidly shifting toward biology-based regeneration. Emerging strategies, including laser-assisted remineralization, electrically enhanced mineral delivery, self-assembling peptides, biomimetic enamel liquids, stem-cell-based therapies, bioactive scaffolds, and gene-focused regeneration, offer promising avenues for rebuilding lost dental tissues rather than merely protecting them. Together, these innovations mark a transition from traditional restorative replacement to minimally invasive, biomimetic, and regenerative treatment strategies, though long-term clinical validation remains essential for their full integration into routine practice.

## Figures and Tables

**Table 1:** Summary of recent research on the use of different agents for management of dental erosion.

Study	Sample/Model	Intervention	Findings	Reference
Cochrane et al. (2020)	Randomised controlled trial	Topical CPP-ACP	Significant reduction in enamel loss compared to control	[6]
Wang et al. (2021)	In vitro pH cycling model	CPP-ACP	Increased microhardness, indicating remineralization	[7]
Lata et al. (2022)	Human enamel in vitro	CPP-ACP and fluoride	Both effective; CPP-ACP showed faster action	[8]
Rodríguez et al. (2023)	In vivo study (12 weeks duration)	CPP-ACP varnish	Significantly reduced dentin surface loss	[9]
Schlagenhauf et al. (2019)	Randomised trial	HAP toothpaste (fluoride-free)	Prevention of caries and erosion in young adults	[10]
Paszynska et al. (2021)	Children aged 6–10	HAP toothpaste (12-week use)	Significant enamel remineralisation without fluoride	[11]
Hao et al. (2021)	Artificial saliva model	fTCP + fluoride mouthrinse	Increased surface hardness, better acid resistance	[12]
Inchingolo et al. (2023)	Clinical trial, 120 patients	TCP-fluoride toothpaste vs. fluoride-only	Superior enamel protection in TCP group	[13]
Souza et al. (2020)	Eroded enamel slabs	CCSP treatment (2×/day)	Reduced mineral loss, formed mineral-rich layer	[26]
Pinto et al. (2021)	Randomised trial, 90 participants	CCSP toothpaste for 8 weeks	Improved enamel smoothness and sensitivity reduction	[27]
Borges et al. (2023)	In vitro acidic challenge model	CCSP + fluoride paste	Reduced enamel surface roughness, improved acid resistance	[28]

## Data Availability

No new data were created or analyzed in this study.

## Abbreviations

The following abbreviations are used in this manuscript:

GERD	Gastroesophageal reflux disease
FDA	U.S. Food and Drug Administration
CPP-ACP	Casein Phosphopeptide Amorphous Calcium Phosphate
TCP	Tricalcium phosphate
HAP	Hydroxyapatite
SHMP	Sodium Hexametaphosphate
STMP	Sodium Trimetaphosphate
